# Complete genome sequence of eight LEE-negative Shiga toxin-producing *Escherichia coli* strains isolated from patients with hemolytic-uremic syndrome

**DOI:** 10.1128/mra.00591-23

**Published:** 2023-12-20

**Authors:** Akiko Kubomura, Kenichi Lee, Makoto Ohnishi, Sunao Iyoda, Yukihiro Akeda, EHEC Working Group

**Affiliations:** 1 Department of Bacteriology I, National Institute of Infectious Diseases, Shinjuku, Japan; 2 Local Public Health Institutes, Japan; University of Maryland School of Medicine, Baltimore, Maryland, USA

**Keywords:** STEC, HUS, LEE negative

## Abstract

Major serotypes of Shiga toxin-producing *Escherichia coli* (STEC) carry a locus of enterocyte effacement (LEE), which is required for attaching and effacing lesion formation. Genome information of LEE-negative STEC is scarce despite their virulence potential. We present the complete genome sequences of eight LEE-negative STEC isolates from hemolytic-uremic syndrome patients.

## ANNOUNCEMENT

Shiga toxin-producing *Escherichia coli* (STEC) is known to be a life-threatening bacterium and is reported constantly as a causal agent of foodborne diseases and infections worldwide. In Japan, approximately 3,000 cases of STEC infection are reported annually (1). Most STEC isolates in these cases carry a 35-kb pathogenicity island termed the locus of enterocyte effacement (LEE), which encodes the ability to induce attaching and effacing lesions on the host intestinal mucosa. It is reported to have different virulence traits according to the presence of LEE, and the presence of LEE differs based on the serotype (2, 3). Although STEC strains that lack LEE (LEE-negative STEC) also can lead to severe human illness and cause hemolytic-uremic syndrome (HUS) (4 – 6), the pathogenicity of LEE-negative STEC is not well understood. In this report, we determined the complete genome sequences of eight LEE-negative STEC strains isolated from patients with HUS in Japan.

From 2008 to 2018, we obtained eight LEE-negative STEC strains from eight patients with HUS. All eight patients appeared to have sporadic STEC infection. These strains were isolated from clinical specimens at the local public health institutes and transferred to the National Institute of Infectious Diseases. The STEC cultures were grown overnight on XM-G agar (Nissui Pharmaceutical, Japan) at 37°C, and the presence of *stx* and *eae* was checked by PCR method in our laboratory (7). The strains carrying *stx* but not *eae* were identified as LEE-negative STEC. Subsequently, the strains were cultured overnight in buffered peptone water (Nissui Pharmaceutical, Japan) at 37°C, and DNA was extracted using the MagMax DNA Multi-Sample Ultra 2.0 kit and the King Fisher Duo Prime (Thermo Fisher Scientific, USA). Genomic DNA libraries were prepared using a QIAseq FX DNA Library Kit (Qiagen, Germany), followed by paired-end sequencing (2  ×  300 mer) using MiSeq (Illumina, USA). Low-quality reads (*Q* scores of <30) were trimmed using Sickle v1.33 (https://github.com/najoshi/sickle). The strains were also sequenced on a PacBio RS II or PacBio Sequel IIe (Pacific Biosciences, USA). Briefly, DNA was sheared 10  to 20  kb using a g-TUBE device (Covaris, USA). Library preparation for PacBio RS II and Sequel IIe sequencing was performed using the SMRTbell Express template preparation kit v1.0 and v2.0, respectively. As for PacBio RS II, size selection with a cutoff value of <15  kb was performed using the BluePippin system (Sage Scientific, USA). The quality control of the raw reads of PacBio RS II and Sequel IIe was performed using SMRT Analysis v2.3.0 and SMRT Link v.10.1.0.119528, respectively. The integrity of each assembled genomic data was confirmed using CheckM v.1.1.2 (8). To obtain complete genomes, hybrid assembly was conducted using Unicycler v. 0.5.0 or Trycycler v 0.5.3 and polished with Polypolish v0.5.0 (9 – 11). Default parameters were used for all software.

The genome information, including the accession numbers, is summarized in Table 1. The complete genomes were annotated using the DDBJ Fast Annotation and Submission Tool (12), followed by manual curation.

**TABLE 1:** Genome metrics and accession numbers of eight HUS patients derived LEE-negative Shiga toxin-producing *Escherichia coli* isolates in Japan^*a*^

Strain name	Isolation year	Source	Serotype	Illumina		PacBio			No. of CDS	G+C content (%)	Chromosome size (bp) (accession no.)	Plasmid size (bp) (accession no.)
				No. of reads (DRA accession no.)	Coverage depth	Sequencing platforms	No. of reads (DRA accession no.)	Read *N*_50_ (bp)				
JNE082640	2008	Stool	OX21:H19	972,386 (DRR489893)	51.2	Sequel IIe	23,876 (DRR489892)	12,281	4,761	50.7	4,923,370 (AP027206)	166,641 (AP027207), 8,121 (AP027208), 7,136 (AP027209), 2,395 (AP027210), 1,552 (AP027211)
JNE120393	2012	Stool	O113:H21	2,537,805 (DRR490207)	129.8	RS II	123,377 (DRR490206)	16,466	4,988	50.8	5,072,830 (AP027246)	160,690 (AP027247), 7,769 (AP027248), 6,774 (AP027249), 3,904 (AP027250), 3,373 (AP027251), 2,257 (AP027252), 1,551 (AP027253)
JNE120442	2012	Blood	O183:H18	2,402,165 (DRR490209)	127.5	RS II	116,511 (DRR490208)	14,735	4,850	51.0	4,967,431 (AP027254)	160,552 (AP027255)
JNE131328	2013	Stool	O113:H21	2,669,163 (DRR490211)	139.7	Sequel IIe	27,130 (DRR490210)	11,764	4,893	50.7	5,018,637 (AP027212)	160,700 (AP027213), 15,094 (AP027214), 6,745 (AP027215), 1,546 (AP027216)
JNE141411	2014	Stool	OgN13:H19	2,658,327 (DRR490213)	139.1	RS II	133,466 (DRR490212)	15,871	4,917	50.7	5,061,037 (AP027256)	173,999 (AP027257)
JNE151685	2015	Stool	O74:H20	2,364,678 (DRR490219)	97.8	RS II	127,596 (DRR490218)	15,675	4,905	50.9	4,968,007 (AP027258)	173,640 (AP027259), 60,729 (AP027260), 6,675 (AP027261)
JNE170426	2017	Urine	OX18:H2	1,213,957 (DRR490215)	59.6	Sequel IIe	28,772 (DRR490214)	10,851	4,936	50.8	5,029,945 (AP027217)	158,915 (AP027218), 86,192 (AP027219), 6,744 (AP027220)
JNE181771	2018	Stool	OX18:H19	2,111,740 (DRR490217)	109.0	Sequel IIe	23,713 (DRR490216)	12,333	4,763	50.8	4,905,183 (AP027156)	131,387 (AP027157), 88,936 (AP027158)

^*a*^ CDS, coding DNA sequences per genome.

## Data Availability

The genome sequences have been deposited in DDBJ/ENA/GenBank under the BioProject number PRJDB14917.
